# TomaNet-6H: towards transparent plant disease detection using a modified CNN with Grad-CAM for tomato leaves

**DOI:** 10.3389/fpls.2026.1970111

**Published:** 2026-10-23

**Authors:** Shubham Roy, Shakir Khan, Neha Gupta, Achin Jain, Arvind Panwar, Fahad Omar Alomary, Abeer Aljohani, Saqib Qamar

**Affiliations:** 1 Department of Information Technology, Bharati Vidyapeeth’s College of Engineering, New Delhi, India; 2 Information Technology Department, College of Computer and Information Sciences, Imam Mohammad Ibn Saud Islamic University (IMSIU), Riyadh, Saudi Arabia; 3 University Centre for Research and Development (UCRD), Chandigarh University, Mohali, India; 4 Galgotias Multi-Disciplinary Research & Development Cell (G-MRDC), Galgotias University, Greater Noida, India; 5 College of Computer and Information Sciences, Imam Mohammad Ibn Saud Islamic University (IMSIU), Riyadh, Saudi Arabia; 6 Department of Computer Science and Information, Applied College, Taibah University, Madinah, Saudi Arabia; 7 Department of Intelligent Systems, KTH Royal Institute of Technology, Stockholm, Sweden; 8 Faculty of Computing and IT (FCIT), Sohar University, Sohar, Oman

**Keywords:** tomato leaf disease classification, deep learning, convolutional neural networks, explainable artificial intelligence (XAI), Grad-CAM, precision agriculture, plant disease detection

## Abstract

Plant diseases pose a critical challenge to agricultural productivity, underscoring the need for rapid and reliable automated detection methods. Manual disease identification is labor-intensive and error-prone, whereas deep learning techniques offer scalable alternatives. This study evaluates five convolutional neural network (CNN) architectures—XceptionNet, DenseNet121, a baseline five-layer CNN, a six-layer CNN, and a proposed custom architecture, TomaNet-6H—for classifying tomato leaf conditions using the PlantVillage dataset. Image preprocessing combines color space transformations and Contrast Limited Adaptive Histogram Equalization (CLAHE), alongside targeted data augmentation to mitigate class imbalance. Experimental results demonstrate that TomaNet-6H achieves a test accuracy of 97.42% and a macro F1-score of 96.60%, while maintaining moderate computational complexity (∼7M parameters) and an inference latency of 68.4 ms per image. Furthermore, Grad-CAM visualizations qualitatively indicate that the network predominantly focuses on symptomatic leaf areas rather than background artifacts. These findings highlight TomaNet-6H as an effective, resource-conscious framework for precision agriculture deployment.

## 1 Introduction

Tomato (*Solanum lycopersicum L.*) is one of the most widely cultivated and economically important horticultural crops worldwide (Moriones and Navas-Castillo, 2000). It plays a significant role in the human diet and contributes substantially to the agricultural economy.Millions of farmers depend on tomato cultivation as an important source of income, and reductions in yield caused by plant diseases can directly affect their livelihoods and disrupt the agricultural supply chain. Maintaining tomato crop health is therefore essential for sustainable production and economic stability. Tomato is also valued for its nutritional composition and diverse culinary applications (Kumar et al., 2020). However, tomato production is continuously threatened by a wide range of fungal, bacterial, viral, and arthropod-related diseases. Fungal diseases such as early blight, caused by Alternaria solani, and late blight, caused by Phytophthora infestans, can cause substantial crop losses, particularly under favorable environmental conditions (Maurya et al., 2022). Other important diseases include leaf spot, leaf mold, bacterial spot, tomato mosaic virus, and tomato yellow leaf curl virus (Novak et al., 2021). Bacterial pathogens like *Xanthomonas perforans* (bacterial spot) degrade plant tissues, affecting the value of the market. The tomato mosaic virus (TMV) and the tomato yellow leaf curl virus (TYLCV) are the most common viral infections. These infections are especially challenging due to their systemic nature and resistance to control measures (Díaz-Pendón et al., 2010). TYLCV is an important concern in warm climates (Varma et al., 2011). Transmission occurs through the whitefly species, *Bemisia tabaci*. In addition, arthropod pests, such as the two-spotted spider mite (*Tetranychus urticae*) cause direct feeding damage and increase vulnerability to secondary infections (Jakubowska et al., 2022). Accurate and timely identification of these diseases is essential for effective disease management. Conventional diagnosis generally relies on visual inspection by farmers or agricultural experts and, where necessary, laboratory-based analysis. Although these approaches can provide useful diagnostic information, they can be time-consuming, labor-intensive, and dependent on the availability and expertise of trained personnel. The variability of disease symptoms can further make visual identification difficult, particularly when different diseases exhibit similar visual characteristics. Consequently, automated image-based disease detection has emerged as an important application of computer vision and machine learning for supporting rapid plant health assessment. From a computational perspective, deep learning, particularly convolutional neural networks (CNNs), has demonstrated strong capabilities for automatically learning discriminative visual features from plant leaf images. Unlike conventional machine-learning approaches that generally require manually designed features, CNNs can learn hierarchical representations directly from input images, making them suitable for complex image-classification tasks. Previous studies using CNN-based approaches have reported high classification performance on benchmark plant disease datasets, including the PlantVillage dataset. However, achieving high classification accuracy is not the only consideration for practical agricultural deployment. Deep CNN architectures can involve a large number of trainable parameters, substantial computational requirements, and longer training or inference times. Such characteristics may restrict their use in resource-constrained environments, including mobile, edge, and field-based agricultural devices. Therefore, an important computational challenge is to develop a disease-classification model that can provide a suitable balance between classification accuracy, generalization, computational complexity, inference speed, and interpretability. Lightweight CNN architectures can potentially address this requirement by reducing computational overhead while retaining effective feature-extraction capability. At the same time, increasing the depth of a network without appropriate architectural mechanisms can make feature propagation and optimization more difficult. Residual connections provide an effective mechanism for improving information and gradient flow between network layers and can facilitate feature reuse. These considerations motivated the development of a compact custom CNN architecture specifically designed for tomato leaf disease classification. In this study, we propose TomaNet-6H, a modified six-layer CNN designed to provide an effective balance between disease-classification performance and computational efficiency. The proposed architecture progressively increases the number of convolutional filters and incorporates a residual connection after the fourth convolutional block to enhance feature propagation and gradient flow. The architecture also employs max-pooling for spatial dimensionality reduction, L2 regularization and dropout to reduce overfitting, and fully connected layers for final multiclass classification. The model is evaluated against established architectures, including XceptionNet and DenseNet121, as well as custom five-layer and six-layer CNN baselines. To improve the reliability of the learning process, image preprocessing and augmentation are employed to standardize the input data and address class imbalance in the tomato disease dataset. The tomato subset of PlantVillage contains 18,160 images belonging to ten disease and healthy-leaf categories. The data are divided into training, validation, and testing subsets, while augmentation is used to increase the representation of underrepresented classes. In addition to conventional classification metrics, the study considers computational efficiency and class-wise performance to provide a more comprehensive evaluation of the proposed architecture. Another important consideration for deploying automated disease-detection systems is model interpretability. High predictive accuracy alone does not necessarily establish whether a CNN is making decisions based on biologically meaningful disease symptoms. To address this issue, Gradient-weighted Class Activation Mapping (Grad-CAM) is incorporated to visualize the regions of tomato leaves that contribute most strongly to the model’s predictions. This provides an interpretable representation of the model’s decision-making process and helps assess whether the network focuses on disease-relevant regions rather than irrelevant background information. The computational design and experimental objectives of this study are therefore motivated by the need for a model that combines accurate classification, computational efficiency, and visual interpretability. The proposed TomaNet-6H achieves a testing accuracy of 97.42% and a macro F1 score of 96.60%, while requiring approximately 7 million parameters and achieving an inference time of 68.4 ms per image. These characteristics indicate its potential suitability for real-time and resource-constrained applications.

This paper is divided into six sections. The first section presents the introduction. Section 2 reviews related work on plant disease classification. Section 3 explains the data set, preprocessing steps, model architectures, and training procedures. Section four discusses the experimental results and performance evaluation. Section 5 highlights the Grad-CAM-based interpretability analysis. Section 6 concludes the study and describes the direction for future work.

## 2 Literature review

Plant disease detection has seen significant progress with the advent of deep learning techniques. Traditional machine learning approaches require manual feature extraction, limiting their ability to generalize across diverse plant disease datasets (Te et al., 2024). As shown in **Table 1**, various DL and ML models have been applied to the detection of plant diseases with promising results. CNN models have demonstrated remarkable success in automating feature extraction and achieving high classification accuracy. Several methods have validated the efficacy of CNN-based models in detecting plant diseases from leaf images. The PlantVillage dataset (Mohanty et al., 2016), has become a benchmark for research on the classification of plant diseases. This dataset includes healthy and infected leaves for multiple crop species, including tomatoes, with various preprocessing techniques such as grayscale conversion and segmented leaf representations. Research using the PlantVillage dataset has shown that CNN-based models can achieve a accuracy of more than 90% in the classification of plant diseases, making it a reliable resource for deep learning applications in agriculture. Several studies have explored CNN approaches for the detection of plant diseases using various architectures. Lin et al. used a CNN with clustering of K means in a cucumber leaf dataset, achieving a recall of 97% and an F1 score of 91%, emphasizing the use of U-Net variants and EfficientNet for segmentation and classification tasks (Lin et al., 2019). Chowdhary et al. proposed a modified U-Net and compared EfficientNet B0, B4 and B7 in the PlantVillage dataset, achieving near-perfect performance with 99. 95% accuracy and F1 score (Chowdhury et al., 2021). Similarly, Aritra Das et al. introduced a lightweight model, XLTLDisNet, that achieved 97. 24% test accuracy, offering a practical solution for real-time applications (Das et al., 2025). Saleem et al. investigated classic architectures such as AlexNet (Sharma et al., 2025), GoogLeNet, and ResNet on a large dataset of 54,306 images, where ResNet-50 performed best with 99% accuracy, complemented by visualization techniques (Saleem et al., 2019). Wang et al. developed Tomato-Det and benchmarked it against YOLO variants using a custom tomato dataset, reporting a high mAP of 92.3% (Wang and Liu, 2024). Chen et al. implemented a CNN based on AlexNet with separable depthwise convolutions, achieving 96% accuracy and reducing parameters while maintaining performance (Chen et al., 2022). Nikitha et al. compared traditional classifiers (SVM, KNN) with CNNs on a soybean dataset, where CNNs outperformed others with 96% accuracy (Nikith et al., 2023). Harakannanavar et al. combined DWT, PCA and GLCM for feature extraction, followed by CNN classification, achieving 99. 09% accuracy in tomato leaf images (Harakannanavar et al., 2022). Yamamoto et al. demonstrated the benefit of super-resolution through SRCNN, improving classification accuracy on low-resolution images to about 90% (Yamamoto et al., 2017). Fuentes et al. utilized real-field images and tested object detection, i.e. AlexNet, GoogLeNet, and ResNet-50, where the latter achieved 85.98% precision (Fuentes et al., 2017). Wang D. et al. proposed OR-AC-GAN for the detection of tomato disease in real environments, achieving 96. 25% accuracy (Wang et al., 2019). Lastly, Durmus et al. evaluated AlexNet and SqueezeNet v1.1, with AlexNet achieving 95. 65% accuracy (Durmuş et al., 2017).

**Table 1:** Literature survey of research works over plant disease detection using DL and ML.

Ref.	Models proposed and used	Dataset	Results	Novelty
(Lin et al., 2019)	CNN with K-means clustering	Cucumber leaf dataset (30 image pairs augmented to 10,000 images)	Precision: 73%, Recall: 97%, F2 Score: 91%	Explored multiple U-Net variants and EfficientNet versions for segmentation and classification
(Chowdhury et al., 2021)	Modified U-Net, EfficientNet B0/B4/B7	PlantVillage dataset (18,161 images)	Accuracy: 99.95%, Precision: 99.94%, Recall: 99.95%	Proposed a modified U-Net and compared EfficientNet B0, B4, and B7
(Das et al., 2025)	XLTLDisNet	PlantVillage dataset (18,161 images)	Train Acc: 99.95%, Val Acc: 97.50%, Test Acc: 97.24%	Introduced a lightweight DL framework for plant disease detection
(Saleem et al., 2019)	AlexNet, GoogLeNet, ResNet	PlantVillage (14 crops, 26 diseases, 54,306 images)	ResNet-50: 99%, GoogLeNet: 98.9%	Detailed visualization techniques for deep models on plant diseases
(Wang and Liu, 2024)	Tomato-Det, R-CNN, YOLOXs, YOLOv5s, YOLOv7-tiny, YOLOv8n	Custom tomato dataset (2000 images)	mAP: 92.3%	Developed and evaluated the Tomato-Det model for tomato disease detection
(Chen et al., 2022)	AlexNet-based CNN with 5-fold cross-validation	PlantVillage dataset (54,306 images)	Accuracy: 96%, Precision: 98%, Recall: 95%, F1: 97%	Used depthwise/separable convolutions to reduce parameters
(Nikith et al., 2023)	SVM, KNN, CNN	Soybean dataset (7000 images)	CNN: 96%, SVM: 76%, KNN: 64%	Compared performance of ML vs DL approaches
(Harakannanavar et al., 2022)	DWT + PCA + GLCM + CNN	PlantVillage with tomato leaves (600 samples)	Accuracy: 99.09%	Hybrid approach combining feature extraction and CNN classification
(Yamamoto et al., 2017)	Super-Resolution CNN (SRCNN)	PlantVillage dataset (54,306 images)	Accuracy: ~ 90%	Improved low-resolution image classification using super-resolution
(Fuentes et al., 2017)	AlexNet, GoogLeNet	Field images (Korean Peninsula)	ResNet-50 Precision: 85.98%	Applied Faster R-CNN, R-FCN, SSD for real-time detection
(Wang et al., 2019)	OR-AC-GAN	Tomato real-environment dataset	Accuracy: 96.25%	Used segmentation and spectral data for disease classification
(Durmuş et al., 2017)	AlexNet, SqueezeNet v1.1	PlantVillage dataset (14 crops, 26 diseases)	AlexNet Accuracy: 95.65%	Reduced computational cost using efficient convolutions

## 3 Methodology

This study uses the PlantVillage dataset for the classification of tomato leaf disease, addressing class imbalance through data augmentation, ensuring 2500 images per class (except those already exceeding 4000). First, we performed pre-processing on the dataset. The images are normalized and augmented. after that, we employ CNN-based architectures, including XceptionNet, DenseNet121, 5-layer CNN, 6-layer CNN, and the proposed CNN (TomaNet-6H), which incorporates residual connections. The hyperparameters are fine-tuned, using a batch size suited for parallel computation, the Adam optimizer for gradient-based optimization. All models are trained on high-performance hardware including GPU (Tesla P100) and TPU (v3-8) to accelerate computation. Apart from various famous performance metrics, ROC curves allow in-depth model comparison and robustness assessment. **Figure 1** shows the complete methodology for the detection of tomato diseases.

**Figure 1:**
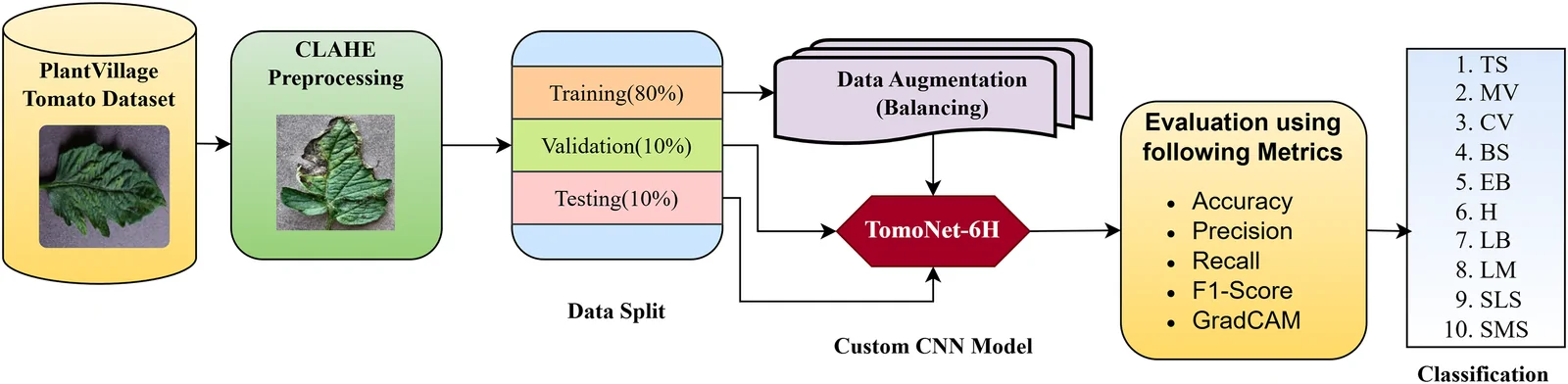
Overall architecture for classification of diseases in tomato leaves.

### 3.1 Dataset details

PlantVillage dataset, sourced from Kaggle (PlantVillage, 2018), has been used in proposed method. It contains 54,305 images across 38 plant disease classes, covering crops such as apple, cherry, corn, blueberry, grape, orange, peach, pepper, potato, raspberry, soybean, squash, strawberry, and tomato. For this research, only the tomato leaf disease subset is used, consisting of 18,160 images in 10 disease classes. The data set is partitioned in a ratio of 80:10:10. Training has 15,523 images, validation has 1,816 images, and testing has 1,821 images. **Figure 2** shows the Sample of dataset.

**Figure 2:**
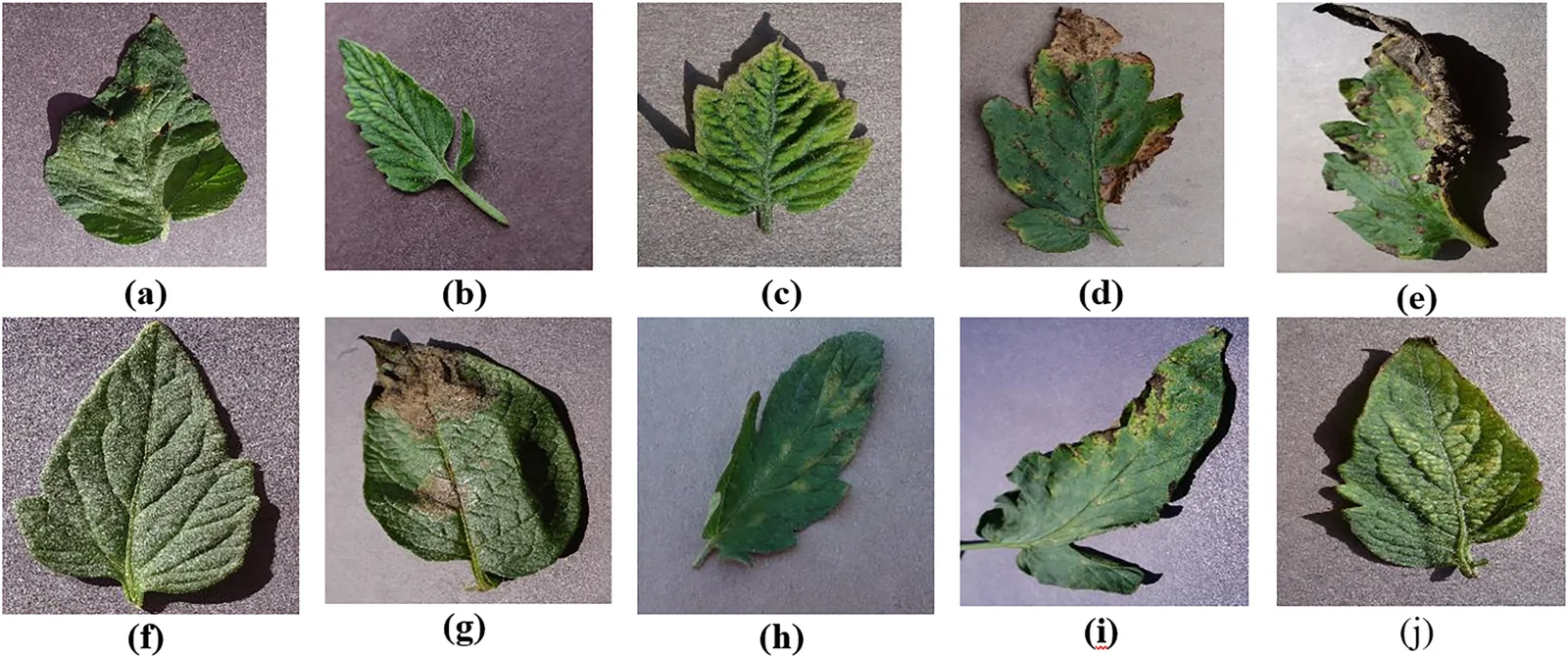
Sample of Dataset: **(a)** Target Spot **(b)** Mosaic Virus **(c)** Yellow Leaf Curl Virus **(d)** Bacterial Spot **(e)** Early Blight **(f)** Healthy **(g)** Late Blight **(h)** Leaf Mold **(i)** Septoria Leaf Spot **(j)** Two-Spotted Spider Mite.

### 3.2 Data preprocessing and augmentation

Data augmentation was employed primarily to address the class imbalance present in the PlantVillage tomato subset and to improve the robustness of TomaNet-6H to common variations in leaf appearance. To prevent data leakage and guarantee valid evaluation, the dataset was strictly partitioned into training, validation, and testing sets prior to applying any augmentation procedures. Consequently, the validation and test sets remained completely unaugmented, consisting exclusively of naturally acquired images. In the original unaugmented training partition, several classes had substantially fewer samples, including Tomato Mosaic Virus (298 images), Early Blight (800 images), and Leaf Mold (761 images). To mitigate this imbalance, controlled offline augmentation was selectively applied only to the underrepresented training classes to expand their representation to approximately 2,500 training samples per class, with the exception of the already dominant Tomato Yellow Leaf Curl Virus. The selected augmentation operations—horizontal flipping, rotation, brightness variation, shearing, zooming, and width/height shifting—were chosen to introduce realistic variations in leaf orientation, scale, illumination, and spatial positioning while preserving the characteristic visual patterns associated with the corresponding disease classes. These transformations modify existing disease-labelled images without generating artificial disease patterns that may not correspond to the actual target pathology, and these augmented instances serve as regularized training variations rather than independent biological samples. Generative AI approaches, including generative adversarial networks (GANs) and diffusion-based image-generation methods, represent an alternative strategy for increasing the diversity of plant disease datasets. Such approaches can potentially generate additional variations in disease appearance, background, illumination, and leaf morphology. However, synthetic images require rigorous quality control and domain validation to ensure that generated samples preserve biologically meaningful disease characteristics and do not introduce unrealistic artifacts or distributional biases. Because the objective of the present study was to evaluate TomaNet-6H under a controlled and reproducible experimental benchmark using the PlantVillage dataset, conventional augmentation was retained for the current experiments, while generative AI-based augmentation is identified as a promising direction for future research. Image preprocessing was performed to standardize inputs and improve model learning. This involved converting images to RGB, resizing to 224×224 pixels for uniformity and efficiency, applying Contrast Limited Adaptive Histogram Equalization (CLAHE) to enhance local contrast of disease features, and using Gaussian blur for noise reduction. Brightness variations were addressed with gamma correction, and an arctangent transformation normalized the pixel intensity distribution. Finally, pixel-wise normalization (mean subtraction and standard deviation division per image) was applied for faster training convergence. The specific augmentation parameters used to increase data variability and simulate diverse imaging conditions are detailed in **Table 2**. The experimental setup used for training and evaluation is summarized in **Table 3**.

**Table 2:** Data augmentation methods.

Augmentation technique	Description
Rescaling	Pixel values normalized to [0, 1] range (rescale=1.0/255.0)
Horizontal Flipping	horizontal_flip=True
Brightness Adjustment	Random brightness variation (brightness_range= [0.8, 1.2])
Rotation	Random rotation up to 90° (rotation_range= 90)
Shear Transformation	Shearing applied with shear_range= 0.2
Zooming	Random zooming within 20% range (zoom_range=0.2)
Width & Height Shifting	Random shifts up to 20%
Fill Mode	New pixel values filled using nearest neighbor interpolation (fill_mode=‘nearest’)

**Table 3:** Hardware and software specifications.

Component	Specification
GPU	NVIDIA Tesla P100, 16 GB (Used: 15.7 GB)
CPU	High-Performance vCPUs
RAM	29 GB
Disk Storage	57.6 GB
Session Duration	12 hours
Operating System	Linux-based Kaggle Environment
Deep Learning Frameworks	TensorFlow, Keras, PyTorch
Python Version	Python 3.x
Jupyter Notebook	Kaggle Notebook

### 3.3 Proposed model: TomaNet-6H

The proposed **TomaNet-6H** is specifically designed for the multiclass tomato leaf diseases. It features a deep and structured CNN architecture optimized for both classification performance and generalization capabilities. The model accepts the resized image as input 224×224 pixels with three RGB channels. It comprises six CNN layers with progressively increasing filter sizes: 64, 64x2, 64x2x2, and 64x2x2x2. Each layer utilizes convolutional kernels 3×3, except for a convolutional layer 1×1 used within a residual block to improve feature refinement and facilitate gradient flow. Each convolutional layer is followed by the Rectified Linear Unit (ReLU) activation function. It has same padding to maintain spatial dimensions, and L2 regularization with λ = 0.001 to mitigate overfitting. To reduce spatial dimensionality and computational complexity, each convolutional block is followed by a 2×2 max pooling layer. A residual connection is introduced after the fourth convolutional block through an Add operation, enhancing the propagation of the features and addressing the problem of vanishing gradients, which supports a more effective training of deeper networks. Final feature vector has the size of 4608. This vector is fed into three fully connected (dense) layers comprising 512, 256, and 10 units, respectively. Softmax is used for classification layer.This output layer produces a probability distribution across the ten target disease classes. Dropout layers are also used to prevent overfitting. The model is trained using Adam Optimizer with a learning rate of 0.0005 and categorical cross-entropy is used as the loss function. Training is carried out with a batch size of 32 for 20 epochs. In general, the TomaNet-6H architecture effectively integrates hierarchical feature extraction, residual learning, and regularization techniques to provide robust and accurate classification of complex images of tomato leaf disease. **Figure 3** and **Algorithm 1** shows the Complete TomaNet-6H Architecture for detection of tomato leaf diseases.

**Figure 3:**
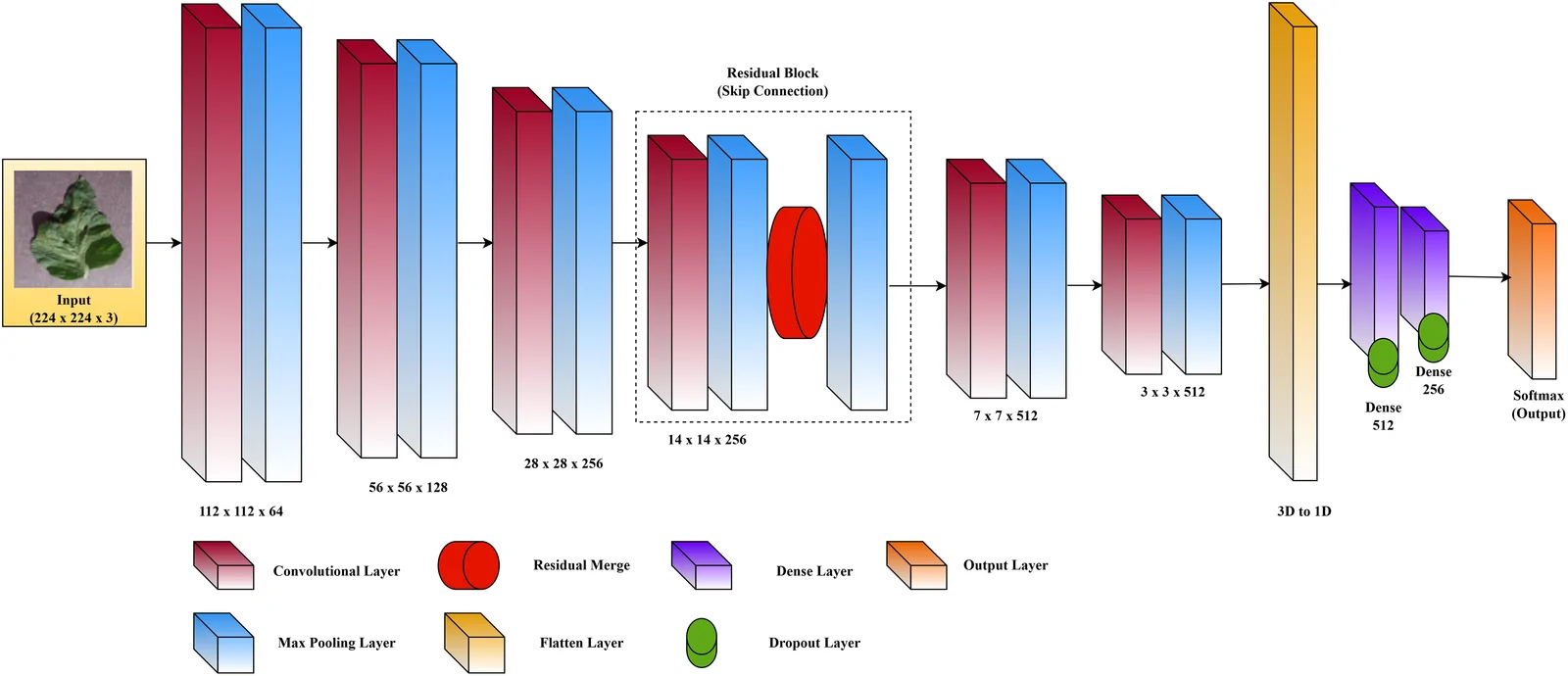
Proposed TomaNet-6H model for the classification of leaf diseases in tomato.

### 3.4 Feature extraction and model components

#### 3.4.1 Feature extraction using convolutional layers

Convolutional layers extract essential spatial and structural features from images of tomato leaves using trainable kernels. Given an input image *X* with dimensions *X*_1_×*X*_2_×*X_C_* and a convolutional kernel *K* with dimensions *k*_1_×*k*_2_×*k_C_*, the output feature map *F* is generated through a convolution operation. The spatial size of the resulting feature map is shown in Equation 1:

(1) $$f=\left(X_{1}-k_{1}+1\right)\times \left(X_{2}-k_{2}+1\right)\times 1$$

The value of each entry *F*[*i*, *j*] in the feature map is computed as shown in Equation 2:

(2) $$F\left[i,j\right]=\sum_{x=1}^{k_{1}}\sum_{y=1}^{k_{2}}\sum_{z=1}^{k_{C}}K\left[x,y,z\right]\cdot X\left[i+x-1,j+y-1,z\right]$$

To introduce non-linearity and enhance learning capacity, the ReLU activation function is applied to the convolution output *u* as shown in Equation 3:

(3) $$\phi_{\text{ReLU}}\left(u\right)=\max\left(0,u\right)$$

This nonlinearity facilitates better gradient propagation by setting negative values to zero.

#### 3.4.2 Residual connection for feature enhancement

To improve gradient flow and feature reuse, a residual connection is incorporated into the fourth convolutional block. Let *X* denote the input feature tensor and let *F*(*X*) denote the output of the convolutional branch. When the spatial or channel dimensions differ, a 1×1 convolution with the required stride is applied to the shortcut branch to obtain a projected tensor *P*(*X*) with dimensions compatible with *F*(*X*). The residual output is then calculated as presented in Equation 4

(4) $$F_{\text{residual}}=\text{ReLU}\left(P\left(X\right)+F\left(X\right)\right).$$

The projection operation ensures that both tensors have identical spatial dimensions and channel depth before the element-wise addition, making the residual operation dimensionally valid.

#### 3.4.3 Pooling layers for dimensionality reduction

Max-pooling layers are applied after each convolutional block to reduce feature map dimensions while retaining key information. The pooling operation *P* is defined as shown in Equation 5:

(5) $$P=\omega_{\text{pooling}}\left(C\right)=\omega_{\text{pooling}}\left(\text{Conv}2\text{D}\left(X,K\right)\right)$$

The dimensions of the pooled output are given as shown in Equation 6:

(6) $$P_{\text{dim}}=\left(\frac{X_{1}+2p-k_{1}}{s}\right)\times \left(\frac{X_{2}+2p-k_{2}}{s}\right)\times X_{C}$$

where *X*_1_×*X*_2_×*X_C_* is the input dimension, *k*_1_×*k*_2_ is the kernel size, *s* is the stride, and *p* is the padding.

#### 3.4.4 Fully connected layers and classification

After convolution and pooling, the feature maps are flattened and passed through fully connected (FC) layers. The operation of an FC layer is given as shown in Equation 7:

(7) $$Y=\sum_{i}w_{i}X_{i}+b$$

where *X* is the flattened input, *w* is the weight matrix, *b* is the bias, and *Y* is the resulting output.

The final output is produced using the Softmax activation function, providing class probabilities is calculated as shown in Equation 8:

(8) $$Z_{i}=\frac{e^{Y_{i}}}{\sum_{j=1}^{l}e^{Y_{j}}}$$

where *Z_i_* is the probability of class *i* among the total *l* classes. Model training has utilized lr:o.0005, loss function:categorical cross-entropy. Training is carried out with a batch size of 32 over 20 epochs. Performance is evaluated using metrics such as accuracy, precision, recall, the F1 score, along with a confusion matrix, and the ROC curve for complete analysis.

ALGORITHM 1

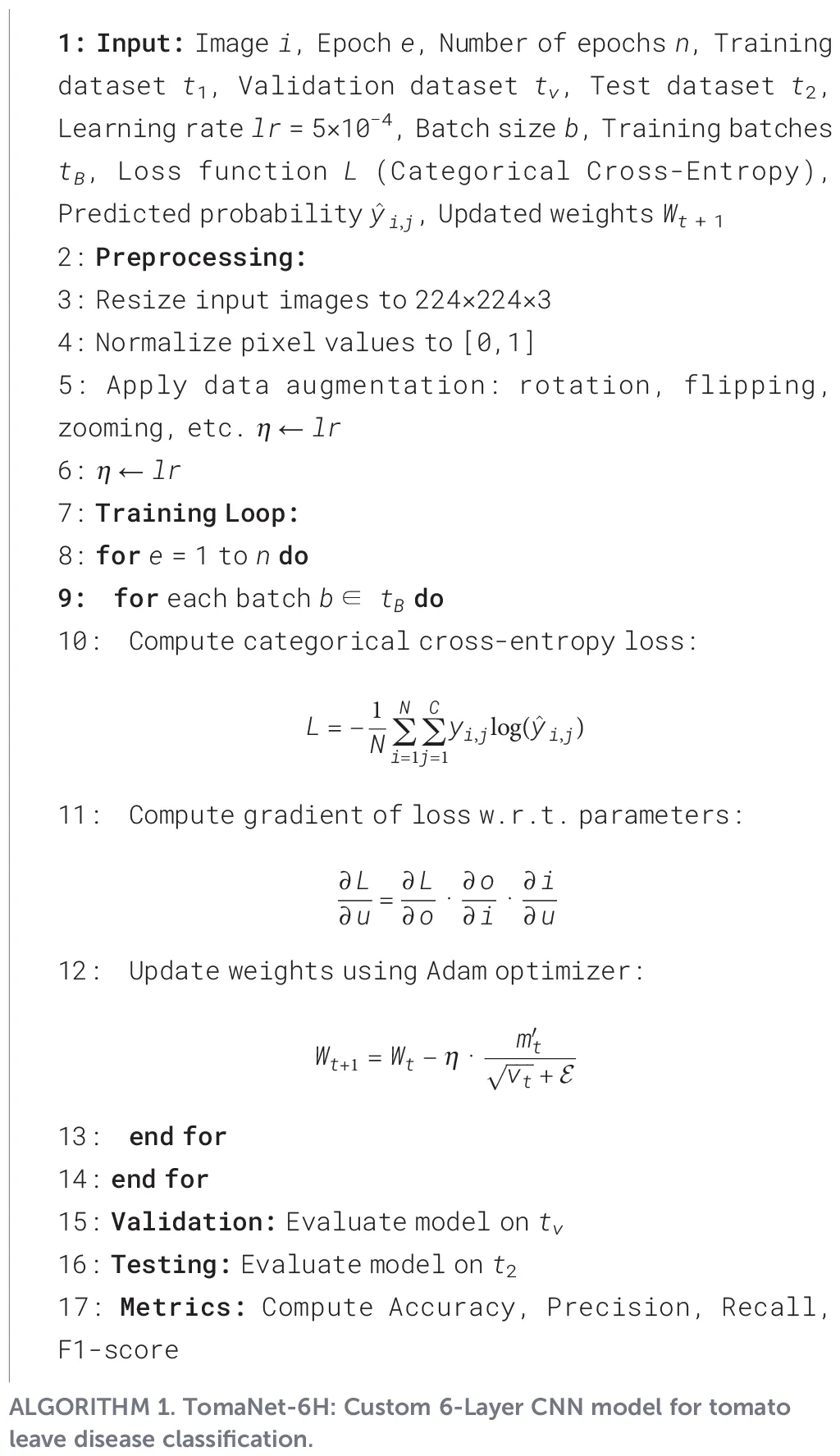

## 4 Result analysis

This section is divided into two parts: Quantitative Analysis and Qualitative Analysis (Explainable AI). The quantitative analysis evaluates the performance of various Convolutional Neural Network (CNN) models using multiple assessment metrics, while the qualitative analysis focuses on the interpretability of these models using visual techniques such as Gradient-weighted Class Activation Mapping (Grad-CAM).

### 4.1 Quantitative analysis

This subsection presents a detailed comparison of training and validation accuracy trends observed over 20 epochs for the deep learning models implemented in this study. The models compared include XceptionNet, DenseNet121, a traditional 5-layer CNN, a standard 6-layer CNN, and the proposed custom 6-layer CNN model. The objective of this analysis is to evaluate the performance of each model in terms of multiple evaluation metrics including Precision, Recall, F1-score, Accuracy, and Training Time. The comparative study aims to identify the strengths and limitations of each architecture when applied to the tomato leaf disease classification task. In addition, class-wise performance is also analyzed to provide insight into how well each model distinguishes between different disease categories. Discussions also include computational efficiency and potential areas for improvement in model design or optimization techniques. The accuracy trends of each model are illustrated in **Figure 4**, providing a comparative analysis of their learning progress. The loss trends for XceptionNet, DenseNet121, 5-layer CNN and 6-layer CNN are shown in **Figure 5**. These visualizations offer valuable information about the model convergence and generalization capability by displaying the variability in the loss values throughout the training process.

**Figure 4:**
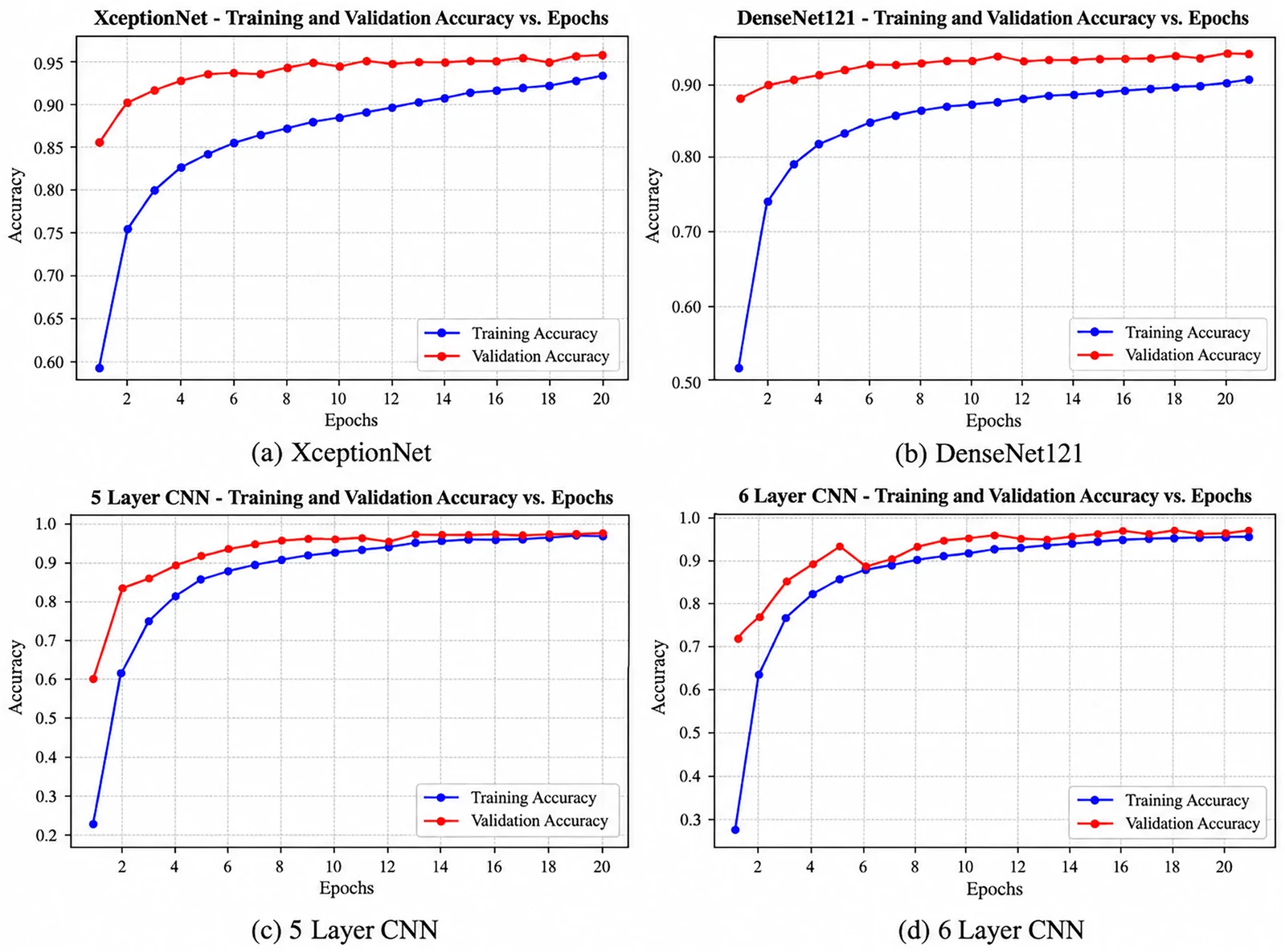
Training and validation accuracy vs. number of epochs for different DL models where: **(a)** XceptionNet, **(b)** DenseNet121, **(c)** 5 Layer CNN, **(d)** 6 Layer CNN.

**Figure 5:**
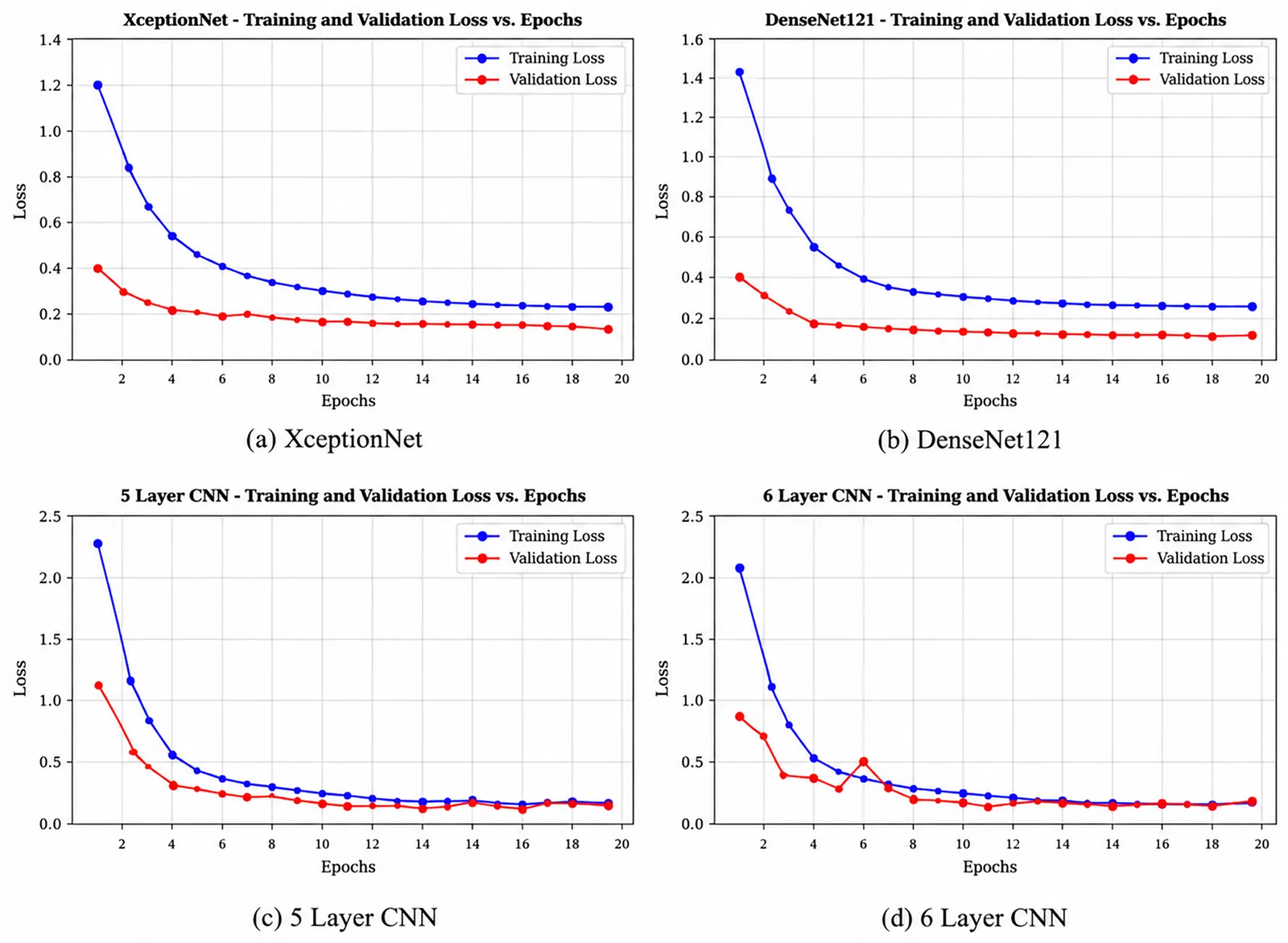
Training and validation loss vs number of epochs for different DL models where: **(a)** XceptionNet, **(b)** DenseNet121, **(c)** 5 Layer CNN, **(d)** 6 Layer CNN.

Furthermore, the loss curve of the proposed custom 6-layer CNN model during the training epochs, as shown in **Figure 6**, highlights its stable learning behavior compared to other models. **Table 4** presents a comprehensive comparison of five deep learning models based on key evaluation metrics, including accuracy, precision, recall, and F1 score. Among all models, the proposed custom CNN (TomaNet-6H) demonstrates the highest performance, achieving a testing accuracy of 97. 42% and an F1 score of 96. 60%, indicating strong generalization and classification capabilities. Although 5- and 6-layer CNNs also perform competitively, TomaNet-6H shows consistent improvements in all metrics evaluated. In contrast, models such as XceptionNet and DenseNet121 exhibit slightly lower robustness in precision and recall. In general, TomaNet-6H strikes an optimal balance between accuracy and computational efficiency, making it the most effective model in this comparative evaluation.

**Figure 6:**
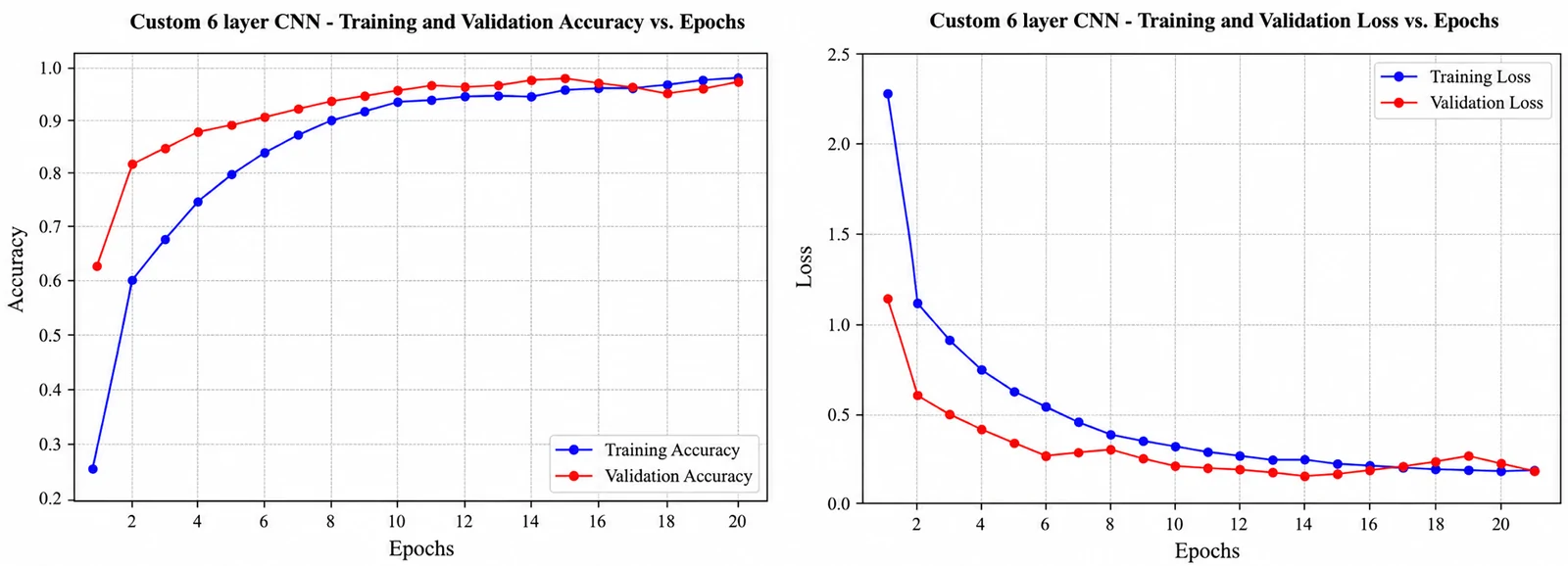
Training accuracy and training loss curves per epoch for Proposed TomaNet-6H.

**Table 4:** Direct experimental comparison of TomaNet-6H with baseline models evaluated under the same experimental conditions.

Model	Accuracy	Precision	Recall	F1-score
XceptionNet	93.47	91.9	90.7	91.26
DenseNet121	95.39	94.1	93.3	93.52
5-Layer CNN	95.72	94.3	94.7	94.41
6-Layer CNN	95.94	95.3	94.7	94.89
TomaNet-6H	97.42	96.3	97.0	96.60

**Figure 7** and **Table 5** provide a detailed comparison of class-wise precision, recall, and F1-score across all evaluated deep learning architectures for the ten tomato leaf categories. The proposed TomaNet-6H consistently achieves the highest or near-highest performance across all metrics, particularly excelling in categories such as Tomato Yellow Leaf Curl Virus (YCV), Bacterial Spot (BS), Early Blight (EB), and Two-Spotted Spider Mite (SMS), with F1-scores approaching or exceeding 0.98. Compared to the benchmark models, TomaNet-6H demonstrates robust generalization across diverse disease symptoms. While DenseNet121 and the baseline 5-layer CNN remain competitive, both exhibit minor performance drops in visually challenging classes such as Tomato Mosaic Virus (MV) and Late Blight (LB). Similarly, XceptionNet shows lower overall consistency, particularly reflected in reduced F1-scores for MV (0.80) and LB (0.84). The balanced precision and recall attained by TomaNet-6H across both dominant and underrepresented categories underscore the effectiveness of its architecture and residual connection in capturing discriminative pathological features.

**Figure 7:**
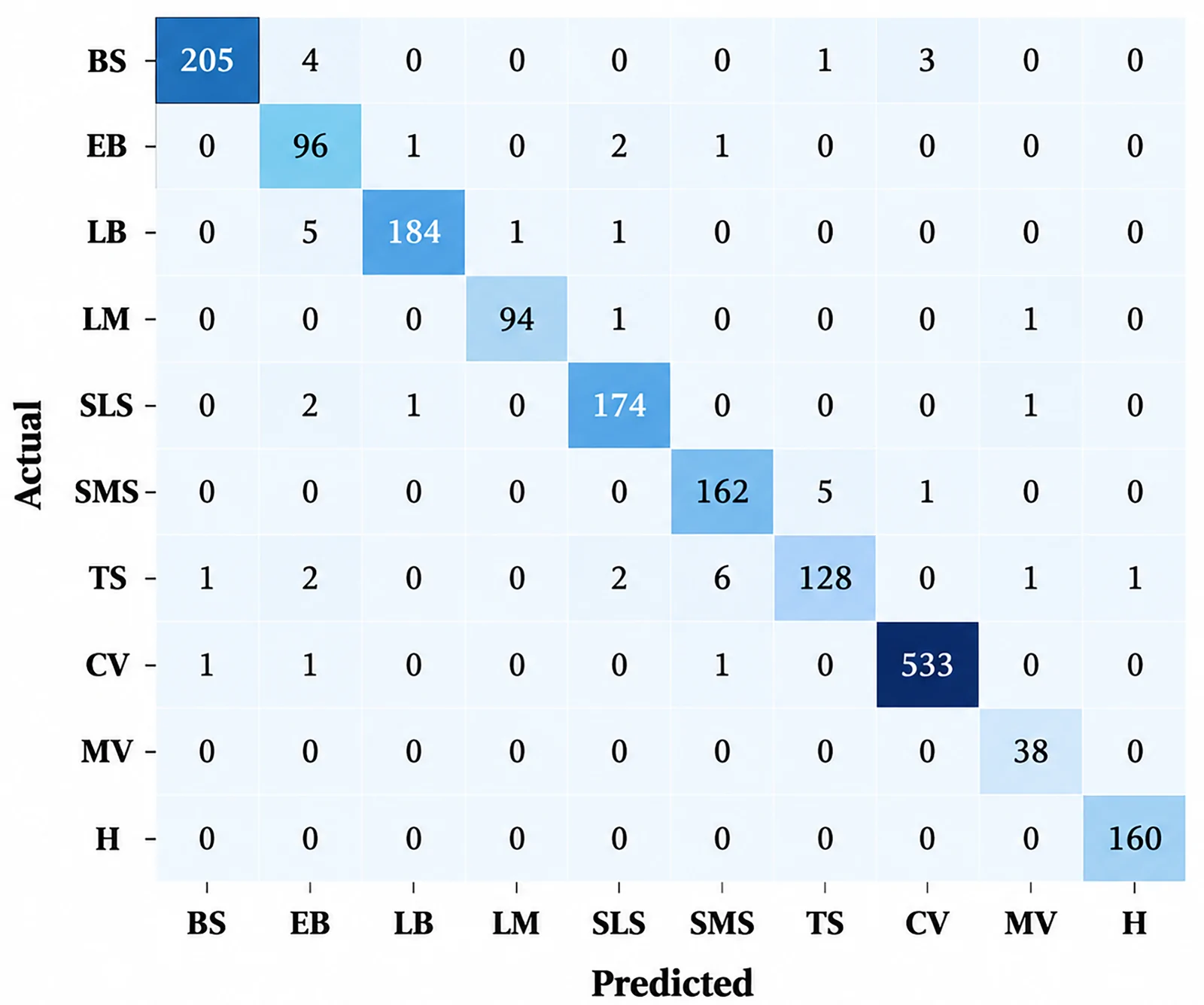
Confusion matrix of proposed TomaNet-6H model.

**Table 5:** Precision, recall, and f1-score for different models across plantvillage tomato classes.

Model	TS	MV	YCV	BS	EB	Healthy	LB	LM	SLS	SMS
Precision										
XceptionNet	0.95	0.82	0.90	0.88	0.93	0.90	0.85	0.99	0.97	1.00
DenseNet121	0.96	0.96	0.94	0.96	0.95	0.93	0.84	1.00	0.90	0.97
5-Layer CNN	0.91	0.87	0.96	0.95	0.97	0.96	0.94	0.99	0.88	1.00
6-Layer CNN	0.91	0.79	0.99	0.97	0.94	0.98	0.96	0.99	1.00	1.00
TomaNet-6H (Proposed)	0.99	0.87	0.99	0.99	0.97	0.95	0.96	0.99	0.93	0.99
Recall										
XceptionNet	0.97	0.79	0.92	0.90	0.94	0.88	0.83	1.00	0.87	0.97
DenseNet121	0.98	0.74	0.96	0.93	0.97	0.91	0.93	0.99	0.95	0.97
5-Layer CNN	1.00	0.82	0.94	0.97	0.94	0.95	0.87	0.99	1.00	0.99
6-Layer CNN	1.00	0.92	0.93	0.94	0.93	0.95	0.87	0.99	0.95	0.99
TomaNet-6H (Proposed)	0.96	0.96	0.96	0.98	0.98	0.96	0.91	0.99	1.00	1.00
F1-Score										
XceptionNet	0.96	0.80	0.91	0.89	0.93	0.89	0.84	0.99	0.92	0.98
DenseNet121	0.97	0.836	0.95	0.945	0.96	0.92	0.88	0.995	0.924	0.97
5-Layer CNN	0.95	0.844	0.95	0.96	0.955	0.955	0.903	0.99	0.936	0.995
6-Layer CNN	0.95	0.85	0.96	0.955	0.935	0.965	0.913	0.99	0.97	0.995
TomaNet-6H (Proposed)	0.975	0.913	0.975	0.985	0.975	0.955	0.934	0.99	0.96	0.995

TS, Target Spot; MV, Tomato Mosaic Virus; YCV, Tomato Yellow Leaf Curl Virus; BS, Bacterial Spot; EB, Early Blight; LB, Late Blight; LM, Leaf Mold; SLS, Septoria Leaf Spot; SMS, Two-Spotted Spider Mite.

- Direct Experimental Comparison: To ensure a fair and consistent evaluation, TomaNet-6H was compared with XceptionNet, DenseNet121, the five-layer CNN, and the six-layer CNN under the same experimental framework. All these models were trained and evaluated using the same PlantVillage tomato leaf dataset, preprocessing procedure, data partitioning, augmentation strategy, evaluation metrics, and experimental settings. Therefore, the performance values reported for these models represent direct experimental comparisons conducted in this study. These results provide the primary basis for assessing the effectiveness of the proposed TomaNet-6H architecture.
- Indirect Experimental Comparison: In addition to the direct experimental comparison, the performance of TomaNet-6H is compared with results reported in previous studies. These comparisons are intended only as contextual benchmarks and should not be interpreted as equivalent to the direct experimental comparison. The reported performance of previous studies may have been obtained using different datasets or dataset subsets, preprocessing and augmentation strategies, training–testing splits, model configurations, hyperparameters, hardware environments, and evaluation protocols. Consequently, differences in reported accuracy and other performance measures may reflect both model characteristics and differences in experimental settings.

The performance of the proposed custom CNN model across various disease classes is summarized in **Table 5**, highlighting its robustness and consistency in classification. The model achieves exceptionally high F1 scores for most classes, with values such as 0.995 for SMS (Two-Spotted Spider Mite), 0.99 for LM (Leaf Mold), and 0.985 for BS (Bacterial Spot), indicating both high precision and recall. Even in relatively harder-to-classify categories like MV (Mosaic Virus), the model maintains a solid F1 score of 0.913, reflecting its reliability across diverse disease types. The corresponding visualization in **Figure 8** reinforces these results by illustrating the trend of the precision, recall, and F1 scores per class, where the consistent height of the bars emphasizes the balanced performance of the model on all target labels.

**Figure 8:**
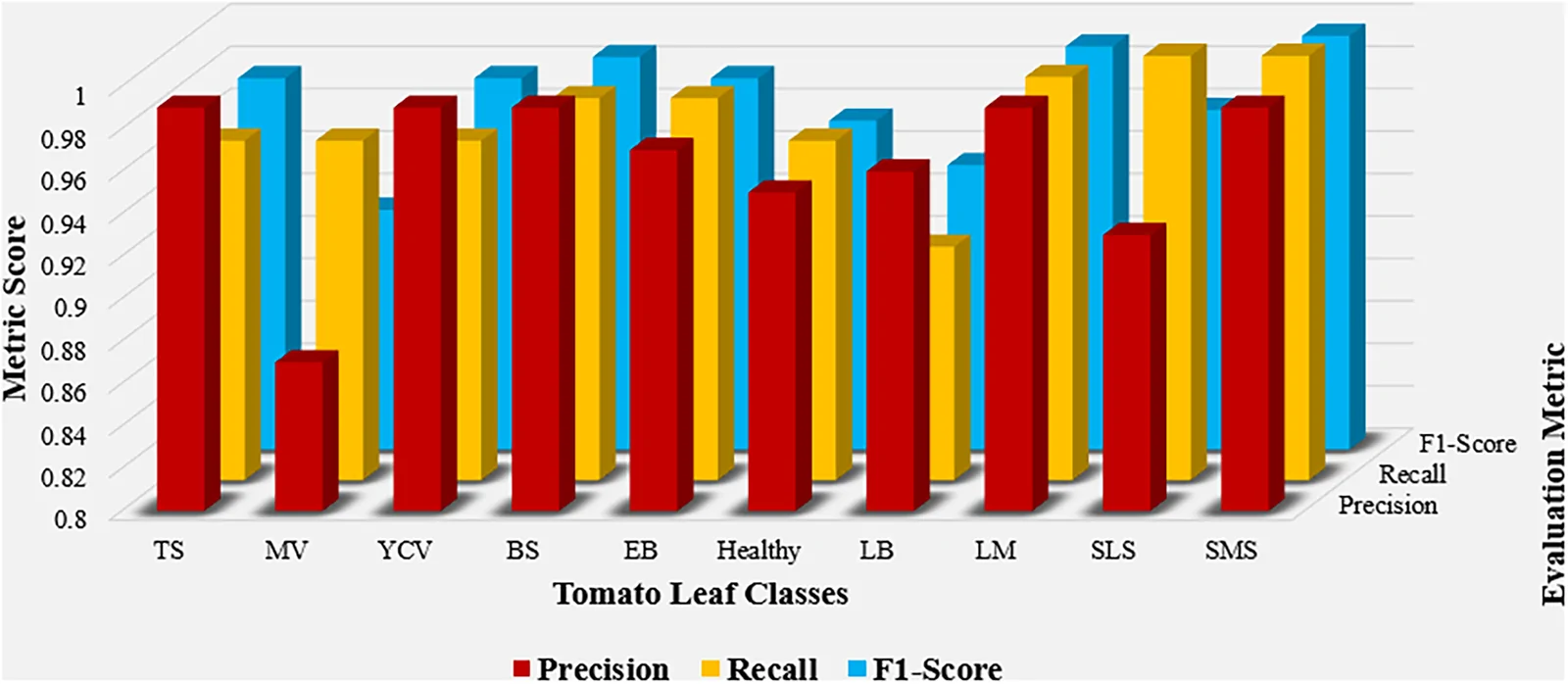
Class wise evaluation metric comparison.

### 4.2 Key insights from the quantitative evaluation

- Difficulty in Classifying Tomato Mosaic Virus (MV): Most models struggled to accurately identify MV, primarily due to the limited number of original training samples before augmentation. Despite this, the proposed Custom CNN achieved the highest recall (0.96) and F1 score (0.913) for this class, indicating its superior ability to learn from limited data.
- Robust and Consistent Performance Across Classes: The Custom CNN (TomaNet-6H) delivered near-perfect precision, recall, and F1 scores in several disease categories, reflecting its effective feature extraction ability and well-balanced class-wise performance.
- Complexity vs. Accuracy Trade-off: Although advanced models like XceptionNet and DenseNet121 performed well in certain metrics, their inconsistent handling of more challenging classes and extended training times make them less ideal than lighter CNN-based architectures, particularly the Custom CNN, for practical deployments.
- Strong Performance from Mid-Sized CNNs: The 5-layer and 6-layer CNNs demonstrated a good balance between classification accuracy and computational efficiency, making them viable options for real-time applications with limited hardware resources.

It is important to mention that certain classes, including Tomato Mosaic Virus, Tomato Early Blight, and Tomato Leaf Mold, had a relatively small number of original training images, 298, 800 and 761, respectively, prior to augmentation. To resolve this class imbalance, data augmentation was applied uniformly, increasing each class to 2500 training samples. Although this approach improved generalization, the abrupt increase in data volume from artificial samples may still fail to represent real-world variability, potentially affecting performance. These insights are further substantiated by **Table 6**, which captures the detailed class-wise performance metrics and further highlights the effectiveness of the proposed custom CNN model.

**Table 6:** Comparison of CNN architectures on tomato leaf disease classification.

Model	Overview	Class-wise performance	Remarks
XceptionNet	Acc: 93.47% F1: 91.26% Time: 5535s	Best: Leaf Mold (F1 = 0.99), Spider Mites (0.98) Weak: Mosaic Virus (Recall=0.79), Late Blight (F1 = 0.84)	Well-generalized with strong major-class performance but high computational cost. Struggles on ambiguous/rare classes. Suitable when accuracy is prioritized over speed.
DenseNet121	Acc: 95.39% F1: 93.52% Time: 2419s	Best: Leaf Mold, Target Spot, YLCV Weak: Mosaic Virus (F1 = 0.84), Late Blight (F1 = 0.88)	Efficient training with strong generalization. Performs well across classes with minor drops on rare cases. A balanced choice between performance and resource usage.
5-Layer CNN	Acc: 95.72% F1: 94.41% Time: 1268s	Best: Spider Mites (F1 = 0.995), Target Spot (Recall=1.00) Weak: Mosaic Virus (F1 = 0.84), Late Blight (F1 = 0.90)	Fast and lightweight with real-time potential. Strong across most classes, but limited depth may hinder learning on complex patterns.
6-Layer CNN	Acc: 95.94% F1: 94.89% Time: 1316s	Best: Spider Mites, Leaf Mold, YLCV Weak: Mosaic Virus (Precision=0.79), Late Blight	Balances training speed and feature depth. Strong generalization with slight drops on Mosaic Virus precision. Slightly slower than 5-layer but more capable.
Custom CNN (TomaNet-6H)	Acc: 97.42% F1: 96.60% Time: 1184s	Best: Target Spot, YLCV, Leaf Mold, Bacterial Spot (F1≥0.975) Weak: Mosaic Virus (F1 = 0.91)	Top performer across all metrics with fastest training among deep models. Residual connections enhance learning. Minimal overfitting and ideal efficiency-performance trade-off. Recommended for practical deployment.

**Table 6** presents a comprehensive comparison of various CNN-based architectures for the classification of tomato leaf disease, highlighting performance metrics, class-wise behavior, and computational aspects. Among all models evaluated, the custom CNN (TomaNet-6H) is the most effective, achieving the highest overall accuracy (97. 42%) and the F1 score (96. 60%) while maintaining the lowest training time (1184.87s) among the deeper models. This model consistently outperforms others in nearly all disease categories, particularly excelling in Target Spot, Yellow Leaf Curl Virus, and Bacterial Spot with F1 scores exceeding 0.975. Its strong generalization ability and lightweight architecture, enhanced with residual connections, enable efficient training and superior class-wise balance. In contrast, although XceptionNet and DenseNet121 perform well in certain classes, they suffer from longer training durations and less robustness in minority or ambiguous classes like Mosaic Virus. The 5- and 6-layer CNNs offer competitive performance with faster training, but exhibit limitations in depth and precision in challenging cases. In general, TomaNet-6H delivers the best trade-off between accuracy, computational efficiency, and class-wise consistency, making it the most suitable model for real-time and resource-constrained deployments.

### 4.3 Explainable AI

To provide transparency and diagnostic insight into model behavior, Explainable AI (XAI) principles were incorporated into the evaluation framework. First, the confusion matrix was examined to identify specific inter-class confusions and assess per-class reliability across challenging visual categories. To provide localized spatial interpretability, Gradient-weighted Class Activation Mapping (Grad-CAM) was applied to the final convolutional layer of TomaNet-6H. Grad-CAM computes the gradients of the target class score with respect to the feature activation maps, generating coarse two-dimensional heatmaps that highlight regions exerting the strongest influence on the classification output. **Figure 9** presents Grad-CAM heatmaps across representative test samples from multiple disease classes. These visual explanations provide qualitative evidence that the network predominantly attends to characteristic symptomatic patterns—such as necrotic lesions, chlorotic margins, and foliar stippling—rather than relying on background artifacts or image borders.

**Figure 9:**
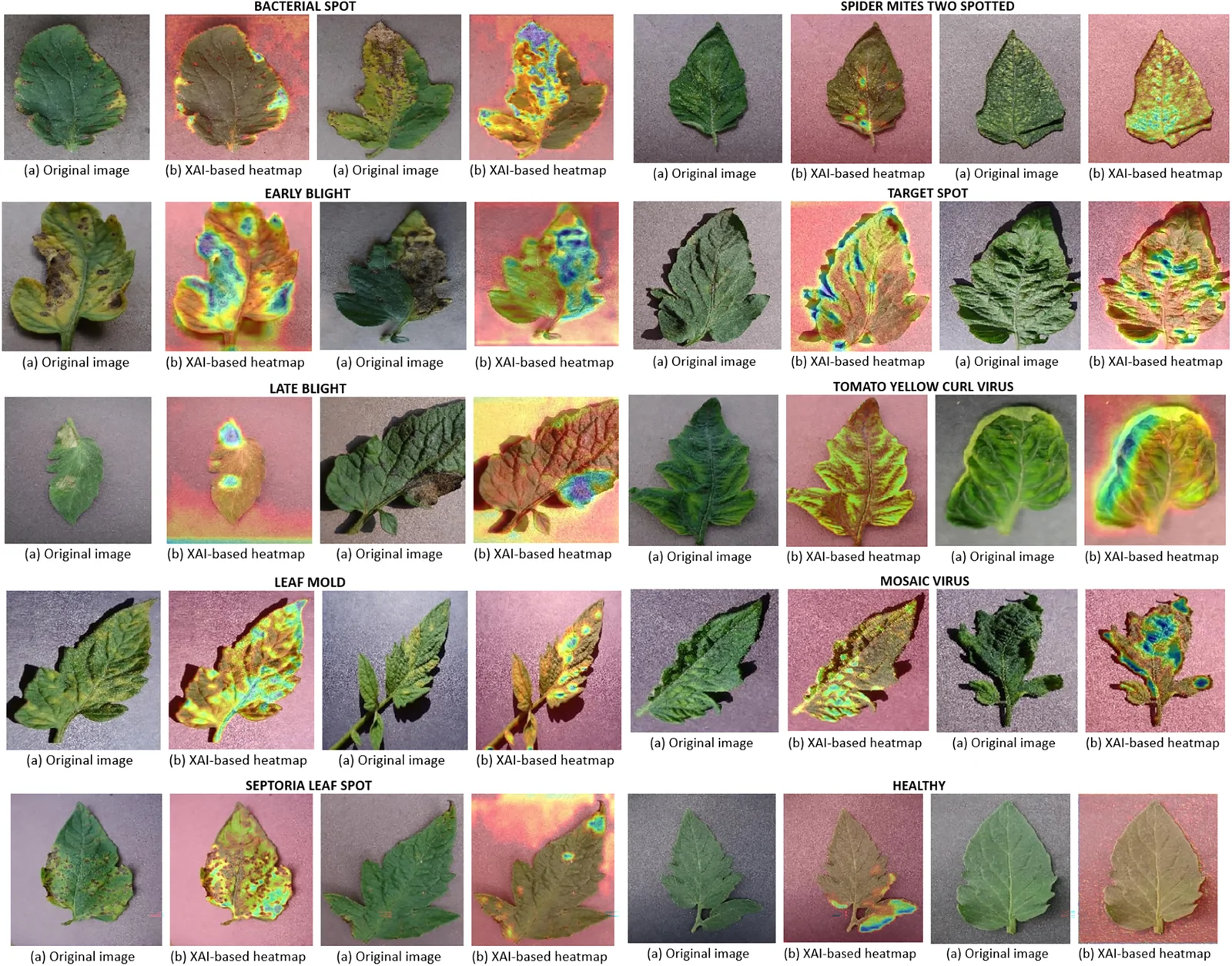
Grad-CAM heatmaps showing salient activation regions across various tomato leaf disease categories for TomaNet-6H.

Importantly, visual alignment between Grad-CAM heatmaps and symptomatic regions serves as a qualitative diagnostic aid rather than definitive proof of explanatory correctness. While the visualizations indicate that the network’s final convolutional features concentrate on disease-affected foliar areas, visual inspection alone cannot establish causal attribution or complete biological fidelity. A formal quantitative evaluation of explanation validity would require benchmarking model saliency maps against pixel-level ground-truth lesion masks annotated by plant pathologists, using metrics such as the pointing game, energy-based localization, or insertion–deletion perturbation curves. Consequently, the present heatmaps should be interpreted as exploratory indicators of intermediate feature salience rather than exhaustive validations of explanation correctness.

### 4.4 Comparison with existing work

To evaluate the performance of the proposed model, TomaNet-6H, a comprehensive benchmarking comparison was conducted using metrics such as macro accuracy, F1 score, inference time, parameter count, and training epochs, as shown in **Figure 10**. The proposed TomaNet-6H achieved the highest macro accuracy of 97.42%, with an F1 score of 96.60% and an inference time of 68.4 ms per image. It was trained over 20 epochs with approximately 7.06 million parameters. In comparison, Aritra Das et al. (2025) attained a similar accuracy of 97.24%, while Altalak et al. (2022) reported 97.2% (Chen et al., 2022). reached 96.00% accuracy with a lightweight AlexNet-based architecture, and (Tm et al., 2018) reported 95.00% (Zhao et al., 2022). achieved 95.20% using an attention-embedded residual-Inception network. These comparisons demonstrate that TomaNet-6H outperforms recent models in terms of accuracy while maintaining efficient inference.

**Figure 10:**
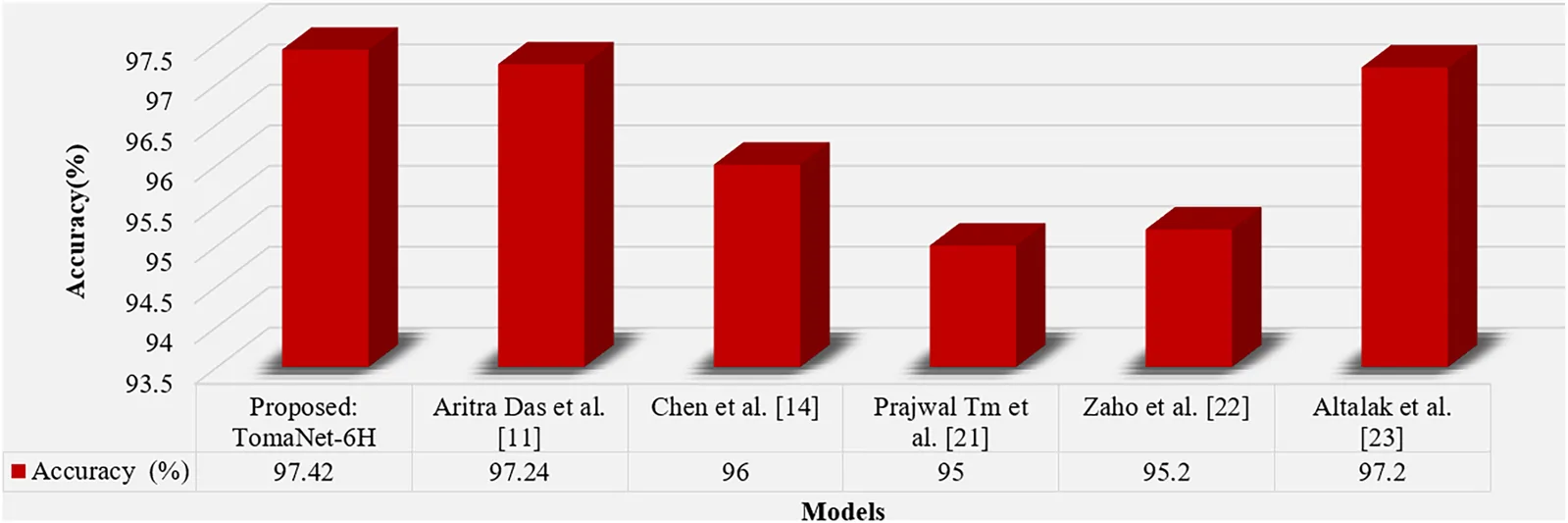
Comparison of TomaNet-6H with earlier work on same dataset.

### 4.5 Discussion

The results obtained from the direct experimental comparison provide a more controlled assessment of the relative performance of TomaNet-6H because the compared architectures were evaluated under the same experimental conditions. In contrast, the comparison with previously published studies should be interpreted cautiously. Although the reported accuracies provide useful contextual information regarding the performance range achieved in the literature, differences in datasets, preprocessing, data splits, augmentation methods, model implementations, hyperparameters, and evaluation environments prevent these values from being considered strictly equivalent. Therefore, the direct experimental results constitute the primary evidence for evaluating the proposed model, whereas published results are used only to provide broader research context. The proposed TomaNet-6H model demonstrates a compelling balance between classification accuracy and computational efficiency. On the PlantVillage dataset, it achieves a macro F1-score of 96.60% and a testing accuracy of 97.42%, outperforming several contemporary CNN architectures. In particular, it maintains efficient inference timing (68.4 ms/image) and relatively low model complexity with approximately 7 million trainable parameters, making it suitable for deployment in real-time or resource-constrained environments such as mobile or edge devices. Compared to deeper architectures like XceptionNet and DenseNet121, which have higher parameter counts and longer training durations, TomaNet-6H achieves comparable or superior performance with significantly lower computational overhead. Against lighter custom CNN baselines (5-layer and 6-layer models), TomaNet-6H demonstrates enhanced feature learning capabilities, attributed to its residual connections and balanced depth, resulting in superior macro precision and recall. Class-wise analysis reveals that the model performs consistently across both major and minor disease classes, excelling in categories such as Target Spot, Tomato Yellow Leaf Curl Virus, Leaf Mold, and Bacterial Spot (F1≥0.975). However, similar to other models, Tomato Mosaic Virus poses a challenge due to visual ambiguity, where TomaNet-6H exhibits a slight dip in performance (F1≈0.91). Compared to previous work, such as (Das et al., 2025) and (Chen et al., 2022), TomaNet-6H provides a more favorable trade-off between accuracy and inference time. Although (Chowdhury et al., 2021) reported a higher macro accuracy (99.84%), their model lacks complete disclosure on trainable parameters and generalization behavior, making direct comparisons limited.

Despite its strengths, the model exhibits important limitations related to dataset dependency. A key constraint of this study is its exclusive reliance on the tomato subset of the PlantVillage repository. While PlantVillage provides a large-scale, standardized benchmark (18,160 images) well-suited for controlled architectural comparison, its images were captured under controlled laboratory settings. These typically feature detached leaves, uniform artificial backdrops, centered leaf orientations, standardized camera distances, and consistent lighting conditions. Deep neural networks evaluated exclusively on such datasets risk learning dataset-specific artifacts and laboratory-conditioned shortcuts rather than invariant pathological markers. Consequently, the reported metrics reflect benchmark performance under controlled acquisition protocols and cannot be assumed to transfer directly to unconstrained field conditions. In real agricultural environments, factors such as dynamic sunlight, deep shadows, complex vegetative and soil backgrounds, wind-induced blur, severe leaf occlusion, and variations across camera sensors present substantial domain shifts that can degrade classification performance. Furthermore, the current training configuration uses a fixed learning rate of 5×10^−4^ without dynamic decay; investigating adaptive learning-rate schedulers represents a valuable direction for future optimization.

To address these challenges, future work will pursue several directions: (i) independent external validation using field-collected datasets (e.g., PlantDoc or newly acquired *in-situ* imagery) across varied crop cultivars and growth stages; (ii) unsupervised domain adaptation (UDA) and transfer learning techniques to bridge the distribution gap between laboratory and open-field imagery; and (iii) generative AI-based synthetic augmentation (such as diffusion models and GANs) alongside conventional transformations to enrich phenotypic diversity while subjecting synthetic samples to domain-specific quality checks before model training.

## 5 Conclusion

Convolutional neural networks (CNNs) have emerged as powerful tools for the automated diagnosis of foliar plant diseases, offering rapid and scalable alternatives to labor-intensive visual scouting. Timely disease detection enables targeted interventions, helping prevent yield losses and supporting sustainable crop management. In this work, we introduced TomaNet-6H, a modified six-layer CNN tailored for the multiclass classification of tomato leaf diseases. Benchmark evaluations on the PlantVillage dataset demonstrated that TomaNet-6H outperforms established deep architectures (XceptionNet and DenseNet121) as well as baseline five- and six-layer CNNs, achieving a test accuracy of 97.42% and a macro F1-score of 96.60%. By incorporating a residual skip connection at the fourth convolutional stage, the architecture enhances feature reuse and gradient propagation while maintaining a compact footprint of approximately 7M parameters and an inference latency of 68.4 ms per image, rendering it practical for deployment on edge and mobile computing devices. To provide transparent insight into model predictions, Grad-CAM heatmaps were examined across representative disease categories, offering qualitative evidence that the network predominantly responds to visually relevant pathological features such as foliar lesions, spots, and chlorosis rather than background artifacts. While the model achieved high classification consistency in controlled benchmark evaluations, future work will prioritize validating TomaNet-6H across unconstrained, open-field datasets (such as PlantDoc and newly captured field imagery) to rigorously evaluate cross-domain robustness under fluctuating ambient illumination, diverse viewing angles, and complex vegetative backgrounds. Furthermore, unsupervised domain adaptation (UDA) methods and generative AI-based synthetic data augmentation will be explored alongside conventional transformations to bridge the lab-to-field distribution gap and enrich phenotypic diversity. In addition, future research will incorporate quantitative XAI evaluation frameworks, including expert-annotated disease masks and pixel-level attribution metrics, to formally evaluate the correctness and causal fidelity of model explanations.

## Data Availability

Publicly available datasets were analyzed in this study. This data can be found here: The dataset used in this study is publicly available as the PlantVillage Dataset?, which can be accessed at: https://www.kaggle.com/datasets/emmarex/plantdisease. The dataset was last accessed on February 16, 2025.
